# Kinase Signaling in Apoptosis Induced by Saturated Fatty Acids in Pancreatic β-Cells

**DOI:** 10.3390/ijms17091400

**Published:** 2016-09-12

**Authors:** Jan Šrámek, Vlasta Němcová-Fürstová, Jan Kovář

**Affiliations:** Division of Cell and Molecular Biology & Center for Research of Diabetes, Metabolism and Nutrition, Third Faculty of Medicine, Charles University in Prague, Ruská 87, Prague 11000, Czech Republic; vlasta.nemcova@lf3.cuni.cz

**Keywords:** c-Jun N-terminal kinase (JNK), protein kinase C (PKC), p38 mitogen-activated protein kinase (p38 MAPK), extracellular signal-regulated kinase (ERK), Akt, fatty acids, pancreatic β-cell, apoptosis, diabetes

## Abstract

Pancreatic β-cell failure and death is considered to be one of the main factors responsible for type 2 diabetes. It is caused by, in addition to hyperglycemia, chronic exposure to increased concentrations of fatty acids, mainly saturated fatty acids. Molecular mechanisms of apoptosis induction by saturated fatty acids in β-cells are not completely clear. It has been proposed that kinase signaling could be involved, particularly, c-Jun N-terminal kinase (JNK), protein kinase C (PKC), p38 mitogen-activated protein kinase (p38 MAPK), extracellular signal-regulated kinase (ERK), and Akt kinases and their pathways. In this review, we discuss these kinases and their signaling pathways with respect to their possible role in apoptosis induction by saturated fatty acids in pancreatic β-cells.

## 1. Introduction

Increased concentrations of fatty acids (FAs) in blood are known to be one of the main factors responsible for pancreatic β-cell death in type 2 diabetes [1,2,3,4,5]. The detrimental potential of FAs has been described for human as well as animal β-cells in vivo and in vitro [1,2,6,7,8,9,10,11,12]. It seems that the toxicity of FAs particularly depends on the degree of their saturation. It was suggested that saturated FAs (e.g., stearic and palmitic acid) induce apoptosis in pancreatic β-cells, whereas the effect of unsaturated FAs (e.g., oleic and palmitoleic acid) on β-cell viability is not entirely clear. It seems that at low concentrations they are well tolerated and are even capable of inhibiting the pro-apoptotic effect of saturated FAs [2,4,5,6,9,13,14,15,16]. Nevertheless, at higher concentrations they might also be pro-apoptotic [17,18,19]. The precise molecular mechanisms of apoptosis induction by saturated FAs in β-cells remain unclear [20]. However, it has been proposed that kinase signaling pathways could be involved [10,21,22,23].

Saturated FAs were shown to induce endoplasmic reticulum (ER) stress in cells including pancreatic β-cells. ER stress was demonstrated to result in activation of signaling pathways starting mainly with three membrane proteins, i.e., inositol-requiring protein 1α (IRE1α), protein kinase RNA (PKR)-like ER kinase (PERK) and activating transcription factor 6 (ATF6). Activation of IRE1α leads to c-Jun N-terminal kinase (JNK) activation by phosphorylation, which further phosphorylates c-Jun. The mentioned signaling pathways primarily participate in the restoration of ER homeostasis. However, if this response fails, apoptosis is induced by mechanisms that are not still completely understood (reviewed in [20,24]).

Kinase signaling pathways are regulated in response to various extracellular physical (e.g., UV radiation, and temperature) and chemical (many agens) stimuli and also in response to various cytokines. They can be involved, depending on cell type, in the regulation of many cellular processes such as proliferation, differentiation, inflammatory response, autophagy, senescence, and also in apoptosis (reviewed in [25]).

In this review, we will discuss kinase signaling pathways with a possible role in apoptosis induction by saturated FAs in pancreatic β-cells. Concerning this, JNK, protein kinase C (PKC), p38 mitogen-activated protein kinase (p38 MAPK), extracellular signal-regulated kinase 1/2 (ERK1/2), and Akt (also known as protein kinase B (PKB) kinase) signaling have been the most extensively studied [26,27,28]. Thus, we will discuss available data on above-mentioned pathways, from both in vitro as well as in vivo experiments using β-cells of animal (mainly rat and murine) and human origin.

## 2. c-Jun N-Terminal Kinase (JNK)

### 2.1. JNK and Its Role in Cell Signaling

JNK is a serine-threonine kinase. It was described in the early 1990s [29,30] when three isoforms were identified, i.e., JNK1, JNK2, and JNK3 (also referred to as stress-activated protein kinase (SAPK)-γ, SAPK-α and SAPK-β, respectively) [31,32,33]. JNK is activated by mitogen-activated protein kinase kinase (MKK) 4 and MKK7 via dual phosphorylation on the tripeptide motif Thr-Pro-Tyr. This tripeptide is located within the activation T-loop in protein kinase subdomain VIII [34]. MKK4 and MKK7 are activated by several MAP kinase kinase kinases (MAP3Ks) as e.g., transforming growth factor-β-activated kinase 1 (TAK1), apoptosis signal-regulating kinase 1 (ASK1), tumor progression locus 2 (TPL2), and mixed-lineage kinases (MLKs) and by some members of the MEKK family. Besides this mechanism, JNK kinase can also be activated by IRE1α protein [35], which represents one of the main signaling pathways of ER stress. It has been showed that ER stress can mediate apoptosis induction by different stimuli including FAs [20,24].

JNK can affect the function of many proteins (reviewed in [36]) including transcription factors (e.g., signal transducers and activators of transcription (STAT), p53, and proteins of forkhead box (Foxo) or ATF family), mitochondrial proteins (e.g., Sab or proteins of B-cell lymphoma 2 (Bcl-2) family), other protein kinases (Akt, p90 ribosomal S6 kinase (p90RSK)), and proteins associated with cell movement (kinesin, doublecortin) or protein degradation (E3 ligase). JNK kinase is mostly involved in mediating the apoptotic response of cells to pro-inflammatory cytokines, and genotoxic or environmental stresses. However, activation of this kinase is also associated with the regulation of cell proliferation, differentiation, survival, and autophagy [34].

### 2.2. JNK in Apoptosis Induced by Saturated Fatty Acids in β-Cells

In Sand rat islets as well as in INS-1 and INS-1E rat β-cells, several authors found that JNK is activated, and mediate apoptosis induction by palmitate or palmitate in combination with high glucose concentrations [22,27,37,38,39,40,41]. We showed that JNK is activated in INS-1E cells in response to pro-apoptotic stearate application (unpublished data). Contrary to previous data, it was also shown that the JNK1 isoform protects against FA/high glucose-induced apoptosis in these cells [42].

Regarding murine pancreatic β-cells, Abaraviciene et al. (2008) [43] as well as Natalicchio et al. (2013) [37] demonstrated in primary islets that JNK activation is involved in palmitate-induced pro-apoptotic signaling. Similar data were obtained in MIN6 and MIN6N8 cells [17,44]. In NIT-1 cells, Yuan et al. (2010) [19] also found that JNK mediates apoptosis induction by palmitate. However, this was not confirmed by Zhang et al. (2007) [26] who documented no effects of apoptosis-inducing palmitate treatment on JNK activation in these cells. Nevertheless, exposure time was only 10 min in their experiment.

In human primary islets, it was demonstrated that JNK activation mediates the pro-apoptotic effects of palmitate [37]. The pro-apoptotic role of this kinase in human islets has also been demonstrated by Aikin et al. (2004) [45]. Authors have also shown that JNK can be inhibited by phosphoinositide 3-kinase (PI3K)/Akt signaling. However, in NES2Y human β-cells, it seems that stearic acid-induced JNK activation, which is associated with the stearic acid-induced ER stress marker immunoglobulin heavy chain-binding protein (BiP), is probably not a key step in apoptosis induction by stearate [46].

It is generally accepted that JNK kinase is activated by saturated FAs-induced ER stress in pancreatic β-cells [20,38].

**Figure 1:**
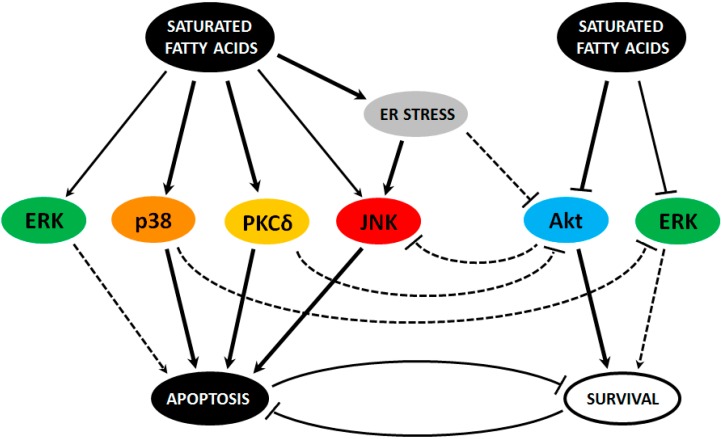
The involvement of c-Jun N-terminal kinase (JNK), protein kinase C (PKC), p38 mitogen-activated protein kinase (p38 MAPK), extracellular signal-regulated kinase (ERK), and Akt kinases and their pathways in apoptosis induction by saturated fatty acids (FAs) in pancreatic β-cells. Solid lines represent relationships with a reasonable probability where bold solid line means more important relationship. Dashed lines represent possible, but less certain, relationships.

To conclude, it has been documented that saturated FAs activate JNK kinase in pancreatic β-cells mainly via ER stress. However, other mechanisms cannot be excluded. It seems that JNK activation mediates apoptosis induction by saturated FAs. Nevertheless, the amount of available data is insufficient to say that this is conclusive. In addition to the process of apoptosis induction, JNK is probably also involved in fatty acid-induced autophagy [47] in pancreatic β-cells. The pro-apoptotic role of JNK has also been documented in osteoblasts and hepatocytes treated with saturated FAs [48,49,50]. JNK seems to be one of the most important kinases in the process of apoptosis induction by FAs in pancreatic β-cells, mainly via ER stress induction (Figure 1).

## 3. Protein Kinase C (PKC)

### 3.1. PKC and Its Role in Cell Signaling

The PKC family of serine-threonine kinases comprises at least ten isoforms that are divided into three groups based on sequence homology and mechanism of activation [51]: (1) conventional PKC (PKCα, β1, β2 and γ); (2) novel PKC (PKCδ, ε, η and θ); and (3) atypical PKC (PKCζ and PKCι/λ). The first two groups are activated by diacylglycerol (DAG) in response to receptor signaling coupled with the activation of phospholipase C. Novel PKC isoforms can be also activated by DAG but are Ca^2+^ insensitive. Atypical PKCs are not activated by DAG and Ca^2+^ but instead in response to receptors that stimulates PI3K leading to activation of phosphoinositide-dependent protein kinase 1 (PDK1).

PKC isoforms have been associated with numerous physiological functions, including secretion and exocytosis, modulation of ion conductance, cell proliferation, and apoptosis or survival [52,53,54]. The PKCδ isoform was the first identified member of the novel PKC group. This isoform was, of the respective PKC isoforms, the most often found to be connected with the process of apoptosis induction via regulation of its down-stream targets, e.g., JNK-STAT1 signaling, Akt, ERK1/2, and p38 MAPK kinases [55,56,57,58].

### 3.2. PKC in Apoptosis Induced by Saturated Fatty Acids in β-Cells 

In the rat INS-1 cell line, it has been shown that PKCδ activation can mediate apoptosis induction by palmitate [23]. Wrede et al. (2003) [28] demonstrated, using the same cell line, the activation of PKCδ by long-chain acyl-CoA. The authors also showed that PKCδ can inhibit Akt kinase. In addition, our unpublished data concerning activation of PKCδ in response to pro-apoptotic stearic acid also suggests that PKCδ is involved in saturated FAs-induced apoptosis in INS-1E cells. In RIN1046-38 rat β-cells, PKCδ activation and translocation from cytosol to the nucleus was found to be necessary for apoptosis induction by saturated FAs (palmitate, stearate) in contrast to unsaturated FAs (palmitoleate, oleate, linoleate) [59]. Similarly, Simon et al. (2008) [60] reported, in rat RINm5F β-cells, that PKCδ kinase was activated in response to low, but still apoptosis-inducing, concentrations of palmitic acid and non-apoptosis-inducing concentrations of stearic acid. The authors suggested that PKCδ mediates apoptosis induction by these FAs. Alcáazar et al. (1997) [61] observed, in Langerhans islets of Wistar albino rats, a translocation of PKC activity (respective isoforms were not tested) from cytosol to cellular membranes after palmitate and high glucose co-application. However, they did not test whether PKC was a mediator of apoptosis induction under these experimental conditions. On the other hand, it seems that in the BRIN-BD11 rat cell line, PKCδ is not required for palmitate-induced apoptosis [9].

Hennige et al. (2010) [12] showed in murine pancreatic β-cells in vivo that overexpression of dominant-negative PKCδ protects against high fat diet-induced β-cell failure through a mechanism that involves inhibition of FA-mediated apoptosis. Furthermore, Qi et al. (2010) [18] documented activation of PKC α and β1 isoforms after exposure to apoptosis-inducing concentrations of palmitate in MIN6 β-cells, which suggests a pro-apoptotic role for the tested isoforms.

With regard to human pancreatic β-cells, there is only one piece of data available. We found no effect on PKCδ activation resulting from treatment with pro-apoptotic concentrations of stearate in NES2Y cells (unpublished data).

Generally, it seems that PKCδ is activated by saturated FAs and it can mediate apoptosis induction by saturated FAs in pancreatic β-cells. However, this role for PKCδ has not been demonstrated in human β-cells, till now (our unpublished data). Nuclear localization might be necessary for pro-apoptotic function of PKCδ. The involvement of PKCδ activation in apoptosis induction in pancreatic β-cells by stimuli other than FAs, such as interleukin 1β (IL-1β) and streptozotocin, has been described [62]. The pro-apoptotic role of PKCδ has been found in a variety of cell types [63,64,65] and is now generally accepted [54]. On the other hand, the α, β, ε, λ, and ζ isoforms of PKC seem to be pro-survival [54]. PKCδ can also inhibit Akt kinase in β-cells. PKCδ seems to be important in the process of apoptosis induction by FAs in pancreatic β-cells, similar to JNK and p38 MAPK (Figure 1). 

## 4. p38 Mitogen-Activated Protein Kinase (p38 MAPK)

### 4.1. p38 MAPK and Its Role in Cell Signaling

To date, four splice variants of the p38 MAPK serine-threonine kinase have been identified: p38α, p38β [66], p38γ [67,68], and p38δ [69,70]. p38 MAPK is mainly activated, as is JNK, by MKK3/6 kinases or, in some specific cell types, by MKK4 kinase. Several MKK kinases (MAP3Ks) have been identified as upstream activators. These include TAK1 [71], ASK1 [72], MLKs [73], and some members of the MEKK family [74,75]. GTP-binding proteins from the Rho subfamily, such as Ras-related C3 botulinum toxin substrate 1 (Rac1), cell division control protein 42 (Cdc42), or Rho [73,76], can contribute to activation of p38 MAPK upstream from MAP3Ks. GTP-binding proteins are activated in response to various extracellular physical (UV light, heat) and chemical (anisomycin) stimuli, and cytokines (tumor necrosis factor α (TNF-α), colony stimulating factor 1 (CSF-1)) (reviewed in [77]).

p38 MAPK can, depending on cell type, regulate activation of various proteins such as transcription factors (CCAAT-enhancer-binding protein C/EBP (CHOP), and activating transcription factor 2 (ATF2)), other protein kinases (MAP kinase-activated protein kinase (MAPKAPK)-2 and -3, and mitogen- and stress-activated protein kinase 1/2 (MSK1/2)), and translation machinery components (eukaryotic translation initiation factor 4E (eIF-4E)) (reviewed in [78]). This signaling pathway can regulate, depending on cell type, different processes including cell cycle, differentiation, inflammation, senescence, autophagy, and apoptosis [77,79].

### 4.2. p38 MAPK in Apoptosis Induced by Saturated Fatty Acids in β-Cells 

It has been showed in rat pancreatic islets and insulinoma cells (RIN) as well as in rat INS-1E cells that p38 MAPK activation mediates apoptosis induction by palmitate [21,37]. Additionally, we have also shown activation of the p38 MAPK pathway (MKK3/6→p38 MAPK→MAPKAPK-2) in INS-1E cells in response to treatment with pro-apoptotic concentrations of stearic acid (unpublished data). 

p38 MAPK activation is involved in palmitate-induced pro-apoptotic signaling in murine primary islets [37,43]. Yuan et al. (2010) [19] obtained similar data in mouse NIT-1 β-cells while Zhang et al. (2007) [26] was unable to confirm these results. They found no effect of apoptosis-inducing concentrations of palmitate treatment on p38 MAPK activation in these cells. However, Zhang et al. (2007) analyzed the FA effect after only 10-min of exposure, compared to 48 h of exposure used by Yuan et al. (2010) [19].

It has been shown in human primary islets that p38 MAPK activation mediates the pro-apoptotic effects of palmitate [37]. Activation of the p38 MAPK pathway (MKK3/6→p38 MAPK→MAPKAPK-2), in response to treatment with pro-apoptotic concentrations of stearate, has been documented in NES2Y human β-cells. It has been shown that this activation could be involved in apoptosis induction by stearic acid; however, this involvement does not seem to play a key role. It has also been shown that activated p38 MAPK probably inhibits the ERK pathway [80].

Concerning a possible connection between p38 MAPK and ER stress signaling in pancreatic β-cells, there is relatively little data available suggesting that p38 MAPK activation functions upstream of ER stress [81].

In summary, it seems that p38 MAPK is activated by saturated FAs and is somehow involved in pro-apoptotic signaling induced by saturated FAs in animal as well as human pancreatic β-cells. Such function of p38 MAPK activation was also demonstrated in rodent and human islets treated by oxidized lipids [82] and pro-apoptotic cytokines [83,84]. The pro-apoptotic role of p38 MAPK signaling has been suggested in other cell types treated with FAs, e.g., coronary artery endothelial cells [85], vascular smooth muscle cells [86], and hepatocytes [87]. p38 MAPK, when activated by saturated FAs in pancreatic β-cells, seems to inhibit ERK1/2 kinase. p38 MAPK seems to be one of the most important kinases in the process of apoptosis induction by FAs in pancreatic β-cells, similar to JNK and PKCδ (Figure 1).

## 5. Extracellular Signal-Regulated Kinase 1/2 (ERK1/2)

### 5.1. ERK1/2 and Its Role in Cell Signaling

ERK1/2 (proteins ERK1 and ERK2 with 85% sequence homology, also known as p42/p44 MAPK) are serine-threonine kinases and are well-known members of the MAPK kinase family. It is a part of the c-Raf→MEK1/2→ERK1/2 signaling pathway, i.e., the ERK pathway. This pathway, depending on cell type, is activated through a variety of different stimuli, including growth factors and cytokines via the relevant receptor tyrosine kinases (RTKs) [88], and ligands of heterotrimeric G protein-coupled receptors [89]. Subsequently, Ras GTPase is activated and recruits c-Raf kinase into a complex where it becomes activated [90]. Then, c-Raf phosphorylates the mitogen-activated protein kinase/ERK kinase (MEK1/2), which in turn activates ERK1/2. c-Raf kinase can be activated, not only by Ras protein, but also by other proteins such as PKC [91], Src [92], and p21-activated protein kinase (PAK) [93].

Depending on the cell type, ERK1/2 kinase regulates the function of numerous substrates across all cellular compartments including several kinases (ribosomal S6 kinase (RSKs), MSKs, and MAP kinase signal-integrating kinases (MNKs)), cytoskeletal proteins (paladin and paxillin), transcription factors (c-Fos, and c-Myc), and membrane proteins (spleen tyrosine kinase (Syk), and calnexin) (reviewed in [94]). The ERK signaling pathway is generally considered to be a key regulator of cell proliferation, however, it may also be involved in the process of differentiation [95], autophagy, senescence, and apoptosis (reviewed in [96]).

### 5.2. ERK1/2 in Apoptosis Induced by Saturated Fatty Acids in β-Cells

Quan et al. (2014) [27] reported that, in the rat INS-1 β-cell line, treatment with apoptosis-inducing concentrations of palmitate led to inhibition of the MEK1/2-ERK1/2 signaling. The authors demonstrated that the signaling had a pro-survival function in INS-1 cells. Guo et al. (2010) [97], using the same cells, also showed ERK1/2 inhibition, even after very brief treatment (10 min) with non-apoptosis-inducing concentrations of palmitate. We have observed activation of the ERK pathway, in INS-1E cells, following exposure to apoptosis-inducing concentrations of stearate (unpublished data). Simon et al. (2008) [60], in RINm5F cells, found activation of this kinase in response to short-term, but not to long-term, treatment with low, but apoptosis-inducing concentrations of palmitic acid and non-toxic concentrations of stearic acids. The authors suggested that persistent ERK1/2 activation via saturated FAs may have pro-survival functions to prevent the toxic effects of late PKCδ activation. Since survival pathways are activated, this may explain why short-period exposure to FA is not enough to induce apoptosis in pancreatic β-cells. 

Plaisance et al. (2009) [98], in murine MIN6 β-cells, showed that ERK1/2 activation is involved in palmitate-induced apoptosis. Activation of ERK1/2 in response to non-apoptosis-inducing concentrations of palmitate was also documented by Fontés et al. (2009) [99] in the same cell line as well as in rat islets. Watson et al. (2011) [100], on the other hand, showed inhibition of ERK1/2 after exposure to pro-apoptotic concentrations of palmitate in MIN6 cells. Abaraviciene et al. (2008) [43] demonstrated ERK1/2 activation in murine primary islets in response to short-term (30 min) treatment with palmitate at apoptosis-inducing concentrations. Longer incubation (24 h) with palmitate had no effect on ERK1/2 activation.

Concerning human pancreatic β-cells, it has been shown in the NES2Y cell line that apoptosis-inducing stearic acid inhibits the ERK pathway, probably via p38 MAPK activation. However, this ERK pathway inhibition does not seem to be essential for stearate-induced apoptosis [80].

A possible connection between ERK1/2 and ER stress signaling in β-cells was also tested, however, with negative results ([81], our unpublished data).

Taken together, the data show that saturated FAs in pancreatic β-cells in some cases stimulate and in some cases inhibit ERK1/2, i.e., the ERK pathway. Reasons for these rather confusing findings are unclear. In the case of ERK1/2 stimulation, it can result in both pro-survival as well as pro-apoptotic effects. Again, these findings are rather confusing. In the case of ERK1/2 inhibition, it seems that the pro-survival effects of ERK1/2 are inhibited. However, we should note that we are dealing with relatively little data. It has been documented that ERK1/2 activation can mediate apoptosis induction by IL-1β or 2,3,7,8-tetrachlorodibenzo-p-dioxin (TCDD) dioxin in pancreatic β-cells and/or rat islets [101,102,103,104]. On the other hand, there are other studies that document the pro-survival functions of the ERK pathway in pancreatic β-cells as well as in mice and human islets [105,106]. In other cell types, ERK1/2 (the ERK pathway) can also have both pro-survival [107] as well as pro-apoptotic [108,109] functions. We should mention that in β-cells treated with saturated FAs, ERK1/2 seems to be inhibited by p38 MAPK. In comparison with the other discussed kinases, ERK1/2 does not play a decisive role in apoptosis induction by FAs in pancreatic β-cells (Figure 1).

## 6. Akt (PKB, Protein Kinase B)

### 6.1. Akt and Its Role in Cell Signaling

There are three known isoforms of Akt kinase, i.e., Aktα (Akt1), Aktβ (Akt2), and Aktγ (Akt3). The pathway leading to the activation of this serine-threonine kinase starts with the interaction of the respective ligands with various RTKs, G-protein coupled receptors, and integrins that activate PI3K. This kinase consequently activates PDK1 through phosphorylation of phosphatidylinositol 4,5-bisphosphate (PIP2) (reviewed in [110]). 

PDK1 further activates Akt kinase, which in turn regulates activation of many substrates involved in cell growth and proliferation, e.g., mammalian target of rapamycin (mTOR) and cyclins (reviewed in [111]), angiogenesis via endothelial nitric oxide synthase (eNOS), (reviewed in [112]), and apoptosis (e.g., FoxO1, Bcl-2-associated death promoter (BAD) or nuclear factor κB (NF-κB) [113,114,115]).

### 6.2. Akt in Apoptosis Induced by Saturated Fatty Acids in β-Cells 

Higa et al. (2006) [10], in the INS-1 cell line, found that Akt activation could mediate apoptosis induction by palmitate. Qin et al. (2014) [23] documented activation of Akt in response to apoptosis-inducing palmitate treatment in the same cell line. We also observed Akt activation due to the application of apoptosis-inducing stearic acid, in INS-1E cells (unpublished data). Fontés et al. (2009) [99] demonstrated in rat islets that cytotoxic palmitate treatment increases Akt phosphorylation. On the contrary, several authors [22,27,40], using similar concentrations and exposure times as in discussed experiments, found that treatment with apoptosis-inducing concentrations of palmitate led to Akt inhibition. They also showed pro-survival function of this kinase in INS-1 cell line. In RINm5F rat β-cells, Simon et al. (2008) [60] showed inhibition of Akt in response to low (0.1 mM) but apoptosis-inducing concentrations of palmitic acid and non-apoptosis-inducing concentrations of stearic acid. The authors suggested that Akt probably has pro-survival function here. A similar role for Akt kinase, in these cells, was also suggested by Nicoletti-Carvalho et al. (2010) [116] who documented that palmitate at apoptosis-inducing concentrations led to inhibition of interleukin-6-activated Akt. However, it is interesting that the application of this acid alone inhibited Akt only very slightly.

Li et al. (2012) [117] demonstrated in murine MIN6 pancreatic β-cells that treatment with cytotoxic concentrations of palmitate led to inhibition of Akt phosphorylation followed by FoxO1 nuclear re-distribution. They demonstrated PI3K/Akt pro-survival and FoxO1 pro-apoptotic functions in these cells. Similar data have also been obtained by other authors [118,119]. Watson et al. (2011) [100] showed that Akt kinase was inhibited after exposure to palmitate at apoptosis-inducing concentrations. Interestingly, Fontés et al. (2009) [99] found in MIN6 cells, that treatment with pro-apoptotic concentrations of palmitate increases Akt phosphorylation 12–18 h after application but not after 24 h. The authors hypothesized that under non-pro-apoptotic conditions, FAs enhances Akt phosphorylation. When either the concentration or the length of exposure is increased, FAs-induced cell death is associated with a secondary decrease in Akt phosphorylation. This idea can be supported by data from Martinez et al. (2008) [17] who demonstrated, in MIN6 cells, that early enhancement of Akt phosphorylation by apoptosis-inducing concentrations of palmitate at 4 h was followed by a decrease at 24 h and was associated with cell death. In the NIT-1 mouse cell line, activation of Akt kinase in response to short-term exposure to pro-apoptotic concentrations of palmitate has been demonstrated [26]. 

With regard to human pancreatic β-cells, the only available data are our unpublished data. We documented Akt kinase inhibition in NES2Y cells in response to apoptosis-inducing concentrations of stearate. However, we did not test whether this inhibition was related to stearate-induced apoptosis.

It was showed that under pathological conditions of chronic activation, ER stress inhibits Akt kinase in pancreatic β-cells [40,120,121]. Akt can also be inhibited by PKCδ [28] and, on the other hand, it can inhibit JNK [45] in β-cells.

Based on the above data, it seems (in spite of some contradictory results, especially in INS-1 cells) that the Akt kinase/pathway is inhibited by saturated FAs and has a pro-survival function in pancreatic β-cells. The pro-survival role of this kinase in β-cells is supported by the findings of Tuttle et al. (2001) [122] that Akt kinase increases β-cell size and mass in C57BL/6J mice and of Aikin et al. (2004) [45] that PI3K/Akt signaling suppresses the pro-apoptotic JNK pathway in human islets. The pro-survival function of Akt kinase/pathway has been documented in other cell types, too (e.g., [123,124]). Additionally, Akt can be inhibited by PKCδ in β-cells.

## 7. Conclusions

According to available data, it seems that JNK, PKCδ, and p38 MAPK kinases and their pathways are activated, JNK via ER stress, by saturated FAs. These kinases probably represent main kinase signaling mediating apoptosis inducted by saturated FAs in pancreatic β-cells. Another important kinase signaling pathway seems to be Akt, which is inhibited by saturated FAs, possibly through ER stress, and has what appear to be pro-survival functions in pancreatic β-cells. The effect of saturated FAs on ERK1/2 and the role of its pathway, in β-cells, after saturated FAs exposure are not completely clear since available data tend to be rather contradictory. The ERK pathway may be activated as well as inhibited by saturated FAs. Similarly, the role of this pathway can be pro-apoptotic as well as pro-survival in pancreatic β-cells. There might be crosstalks between some of the discussed pathways (Figure 1).

## Abbreviations

ASK1	apoptosis signal-regulating kinase 1
ATF2	activating transcription factor 2
ATF6	activating transcription factor 6
BAD	Bcl-2-associated death promoter
Bcl-2	B-cell lymphoma 2
BiP	immunoglobulin heavy chain-binding protein
Cdc42	cell division control protein 42
CHOP	CCAAT-enhancer-binding protein C/EBP
CSF-1	colony stimulating factor 1
DAG	diacylglycerol
eIF-4E	eukaryotic translation initiation factor 4E
eNOS	endothelial nitric oxide synthase
ER	endoplasmic reticulum
ERK1/2	extracellular signal-regulated kinase 1/2
FA	fatty acid
Foxo	forkhead box
IL-1β	interleukin 1β
IRE1α	inositol-requiring protein 1α
JNK	c-Jun N-terminal kinase
MAPK	mitogen-activated protein kinase
MAPKAPK	MAP kinase-activated protein kinase
MEK1/2	mitogen-activated protein kinase/ERK kinase
MKK	mitogen-activated protein kinase kinase
MLKs	mixed-lineage kinases
MNKs	MAP kinase signal-integrating kinases
MSK1/2	mitogen- and stress-activated protein kinase 1/2
mTOR	mammalian target of rapamycin
NF-κB	nuclear factor κB
PAK	p21-activated protein kinase
PERK	protein kinase RNA (PKR)-like ER kinase
PIP2	phosphatidylinositol 4,5-bisphosphate
PI3K	phosphoinositide 3-kinase
PDK1	phosphoinositide-dependent protein kinase 1
PKB	protein kinase B (also known as Akt kinase)
PKC	protein kinase C
p90RSK	p90 ribosomal S6 kinase
Rac1	Ras-related C3 botulinum toxin substrate 1
Raf	rapidly accelerated fibrosarcoma
Ras	rat sarcoma
RTKs	receptor tyrosine kinases
SAPK	stress-activated protein kinase
STAT	signal transducers and activators of transcription
Syk	spleen tyrosine kinase
TAK1	transforming growth factor-β-activated kinase 1
TCDD	2,3,7,8-tetrachlorodibenzo-p-dioxin
TNF-α	tumor necrosis factor α
TPL2	tumor progression locus 2
